# One-Step Preparative Separation of Phytosterols from Edible Brown Seaweed *Sargassum horneri* by High-Speed Countercurrent Chromatography

**DOI:** 10.3390/md17120691

**Published:** 2019-12-09

**Authors:** Menglu Xia, Chunping Liu, Lei Gao, Yanbin Lu

**Affiliations:** 1 Key Laboratory of Aquatic Products Processing of Zhejiang Province, Institute of Seafood, Zhejiang Gongshang University, Hangzhou 310035, China; 2 Hangzhou Nafen BioChem Corporation, Hangzhou 310008, China

**Keywords:** *Sargassum horneri*, saringosterol, fucosterol, phytol, high-speed counter-current chromatography, preparative separation

## Abstract

*Sargassum horneri*, a sargassaceae brown alga, is one of the main species in the subtidal seaweeds flora extensively distributed in the Yellow and East China Sea. It has been proven that the phytosterols are an important class of bioactive substances in *S. horneri*. In this work, a counter-current chromatography approach is proposed for preparative separation of phytol and two analogue sterols from a crude extract of *S. horneri*. A two-phase solvent system composed of *n*-hexane-acetonitrile-methanol (5:5:6, *v*/*v*) was selected and optimized. The effects of rotary speed and flow rate on the retention of the stationary phase were carefully studied. Under the optimum conditions, phytol and two analogue sterols, fucosterol and saringosterol, were baseline separated, producing 19.8 mg phytol, 23.7 mg fucosterol, and 3.1 mg saringosterol from 300 mg of crude *S. horneri* extract in one-step separation. The purities of three target compounds were all above 85%. The structures of phytol and two sterols were identified by nuclear magnetic resonance spectroscopy.

## 1. Introduction

**Figure 1:**
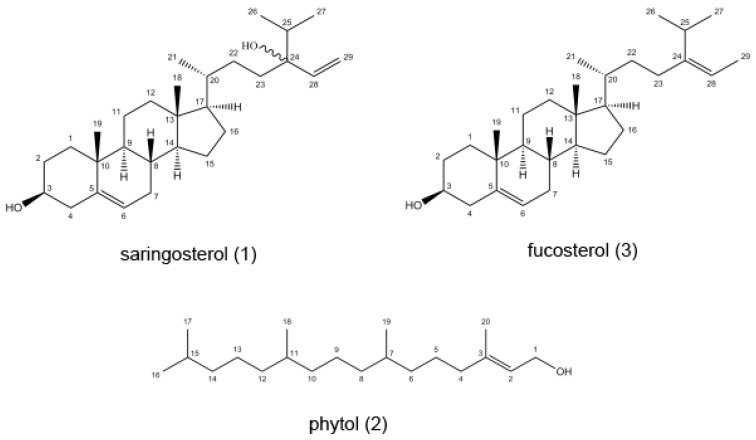
Chemical structures of target compounds separated from *S. horneri.*

*Sargassum horneri*, a type of brown algae, is widely distributed in China, Korea, and Japan [1,2]. It is suitable for growing in the sea area which has a strong combination of high water transparency and abundant nutrient salt. *S. horneri* has been widely used as an edible alga and has various health benefits, such as protective role on bacteria, anti-proliferative effects on cancer cells, anti-inflammatory and antioxidant activity [3,4,5]. Due to the high content of polysaccharides, there are many studies on polysaccharides, and many achievements have been accomplished [6,7,8]. While, as another abundant compounds family present in *S. horneri*, the development of sterol-related health care products is relatively slow. In fact, studies have shown that phytosterols have a wide range of biological activities and have important properties such as antibacterial, antiviral, antioxidant, and so on [9,10,11,12,13,14]. Recently, fucosterol and saringosterol (see Figure 1) were evaluated for their effects on LXR-mediated transactivation and target gene expression in six cell lines. The exciting result was that 24(*S*)-saringosterol acts as a novel selective LXRβ agonist. This study also demonstrated that phytosterols in *Sargassum* family contributed to the well-known antiatherosclerotic function [9]. However, due to the similar chemical structure and low polarity, traditional purification procedures always suffer from tedious, time-consuming sample pretreatment, re-chromatographic steps, and low yields.

High-speed countercurrent chromatography (HSCCC) is a continuous liquid–liquid distribution chromatographic technique that does not have a solid matrix and relies solely on centrifugal force and its own gravity to retain the stationary phase [15]. This technology achieves the purpose of efficient separation with a two-phase solvent system. Comparing with the traditional liquid-solid chromatography techniques, it benefits from great advantages: eliminating the complications derived from the solid support matrix, such as irreversible adsorptive sample loss and deactivation, tailing of solute peaks, and contamination. CCC has many unique merits, such as higher material recovery and product purity, shorter separation time, a larger scale of sample-loading and a wider range of feasible two-phase solvent systems [16,17]. Therefore, CCC has been successfully applied for the isolation of various bioactive compounds from natural origins [18,19,20,21,22]. 

The purpose of this study, therefore, is to develop an efficient CCC method to preparative isolation of phytosterols from the crude extract of *S. horneri.* For this purpose, a two-phase solvent system composed of *n*-hexane-acetonitrile-methanol (5:5:6, *v*/*v*) was selected and optimized. The effects of rotary speed and flow rate on the retention of the stationary phase were carefully studied. Under the optimum conditions, phytol and two analogue sterols, fucosterol and saringosterol (see Figure 1), were baseline separated from 300 mg of the crude *S. horneri* extract in a one-step separation. The chemical structures of target compounds were identified by nuclear magnetic resonance spectroscopy. 

## 2. Results and Discussion

### 2.1. HPLC Analysis of the Crude Extract

In the present work, the crude *S. horneri* extract was obtained by a heat reflux and saponification process. Saponification efficiently removes chlorophylls and lipids, which might result in interference in the spectrophotometric assay of phytosterols [21]. The obtained crude sample was stored in a refrigerator (−20 °C) for the subsequent CCC separations.

**Figure 2:**
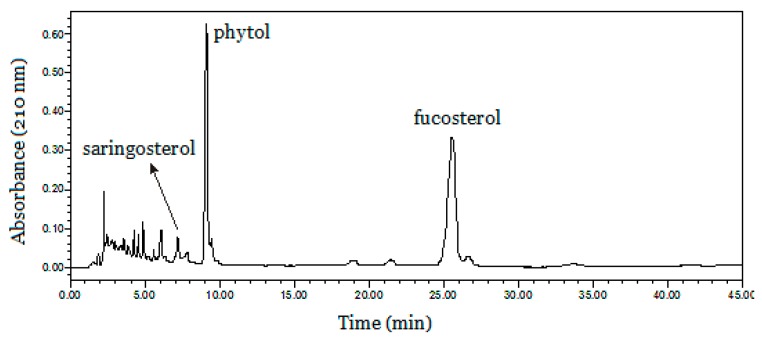
HPLC analysis of the crude *S. horneri* extract.

Subsequently, a suitable HPLC method for analysis of the crude sample and peak fractions of HSCCC was optimized. In order to select an appropriate elution system for HPLC analysis of the crude *S. horneri* extract, different kinds of solvent systems were employed. The results revealed that when methanol and acetonitrile (3:7, *v*/*v*) were used in the isocratic elution mode and the flow rate was 0.8 mL min^−1^, the target compounds could be well separated from other components. As shown in Figure 2, the HPLC chromatogram of the crude extract shows several compounds where the content of target compounds **1**, **2** and **3** is 1.6, 8.9, and 10.1%, respectively, based on the HPLC peak area percentage.

### 2.2. Selection of Two-phase Solvent System for CCC

The crude *S. horneri* extracts have lipophilic physical properties and are soluble in non-polar solvent. Therefore, *n*-hexane–acetonitrile solvent system was selected to separate the target compounds due to it satisfying the requirements. To achieve a successful separation using HSCCC, the partition coefficient (*K*) is the most important parameter for the selection of the solvent system, which should be in the range of 0.5–2.0 to get an efficient separation and a suitable run time. Small *K* value usually results in poor resolution, while a large *K* value tends to get a broader peak and more dilute peak fractions due to a longer elution time. In the present study, different kinds of solvent systems were tested. As seen in Table 1, when the solvent system *n*-hexane-acetonitrile (1:1, *v*/*v*) was used, compounds **2** and **3** gave very large *K* values, implying broader peak and longer separation time, which was not ideal for rapid CCC separation. When *n*-hexane-acetonitrile-dichloromethane (10:7:3, *v*/*v*), *n*-hexane-acetonitrile-dichloromethane (8:7:3, *v*/*v*), and *n*-hexane-dichloromethane-methanol-acetonitrile (10:3:2:5, *v*/*v*) were used, the targets produced appropriate *K* values. However, the separation factor should also be taken into consideration. As shown in Table 1, the peak resolution between compound **1** and compound **2** was extremely fine (*α* > 1.5) for all the studied systems, indicating that the saringosterol was easily separated from phytol. On the contrary, the separation factor between compound **2** and compound **3** was close to 1.0 for these three systems. Therefore, it was almost impossible to achieve baseline separation in a single CCC run. Consequently, according to the literature [22], the biphasic liquid systems composed of *n*-hexane-acetonitrile-methanol at different ratios have been under evaluation. Data shown in Table 1 indicates that this series of biphasic liquid systems can produce appropriate *K* values for all targets. Moreover, the separation factor of phytol and fucosterol was greatly improved. When the two-phase solvent system composed of *n*-hexane-acetonitrile-methanol (5:5:6, *v*/*v*) was used, the peak resolution between phytol and fucosterol was better. Therefore, this solvent combination was selected for the subsequent CCC separations.

### 2.3. Studies on the Retention of the Stationary Phase 

CCC is a unique liquid–liquid partition chromatography without the use of solid support matrix. Thus, the retention of the stationary phase (*S*_F_) is a very important parameter in this chromatographic method. The higher the retention of the stationary phase, the better the peak resolution. Generally speaking, the value of *S*_F_ is affected by various factors such as the physical properties of the two-phase solvent system, column geometry, revolutional speed of the column, and the flow rate of the mobile phase [16,17]. Because of the complex hydrodynamic behavior of the two phases in the column, retention of the stationary phase under a given set of conditions is best determined by actual experimentation.

**Figure 3:**
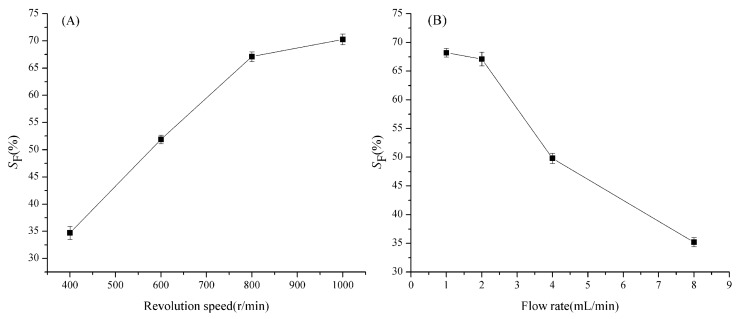
(**A**) Effect of rotating speed on the stationary phase retention (flow rate: 2.0 mL min^−1^). (**B**) Effect of flow rate on the stationary phase retention (rotary speed: 800 rmp).

In the present studies, with a two-phase solvent system composed of *n*-hexane-acetonitrile-methanol (5:5:6, *v*/*v*), the effects of applied centrifugal-force field and the flow rate of mobile phase on the value of *S*_F_ (retention of the stationary phase, %) were under systematic considerations. The results are summarized in Figure 3, where the percentage retention of the stationary phase was plotted against the applied revolutional speeds (Figure 3A), as well as the mobile phase flow rates (Figure 3B). In general, retention of 50% is considered to be satisfactory. The retention curves in Figure 3 show that *S*_F_-values are almost over 50% under each experimental condition, indicating that the selected two-phase solvent system is very suitable for a preparative HSCCC method. In addition, both reducing the flow rate and expediting the rotary speed can increase the retention of the stationary phase. Thus, when the revolutional speed was 1000 rpm, and the flow rate of the mobile phase was 1.0 mL min^−1^, the value of *S*_F_ could get to its maximum. However, longer elution time and broader peaks could be caused by a lower flow rate. In addition, considering the mechanical aspect of the instrument’s rotor, the flow rate of the mobile phase was set at 2.0 mL min^−1,^ and the rotary speed of the column was performed at 800 rpm during all separations.

### 2.4. HSCCC Separations 

**Figure 4:**
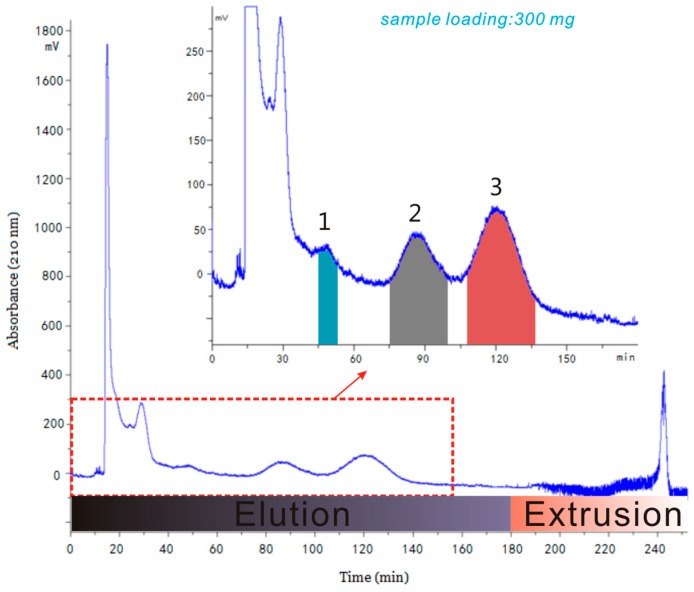
The chromatogram of HSCCC separation of target compounds from *S. horneri* extracts.

Figure 4 illustrates the preparative HSCCC separation of target components from 300 mg of crude *S. horneri* extract with the biphasic solvent system composed of *n*-hexane-acetonitrile-methanol (5:5:6, *v*/*v*). The CCC separation was performed on the 140 mL-capacity instrument. In order to keep the satisfactory peak resolution, the flow rate was set at 2.0 mL/min, and rotary speed was set at 800 rpm. After hydrodynamic equilibrium was reached, 10.0 mL sample solution (samples were dissolved in a 1:1 mixture of upper and lower phase) containing 300 mg crude extract was manually injected into the sample loop. The effluent from the tail end of the separation column was continuously monitored with a UV detector at 210 nm. Under the above optimum conditions, satisfactory stationary phase retention was obtained (*S*_F_ = 67.1%). In order to save solvents and time, the elution-extrusion separation mode [23,24] was employed, and the extrusion process was employed after 180 min: Two immiscible phases leave the column during the EECCC separation. The shading scheme on the *x*-axis (Figure 4) shows the positions of the different elution-extrusion stages: elution and extrusion. A clear jump of the UV signal was observed when the stationary phase replaced the mobile phase in the UV detector. Dramatic noise was also observed. It is presumed that the poor UV detection was mainly due to micro-droplets of the mobile phase (lower phase) staying in the detector cell and perturbing the light beam.

**Figure 5:**
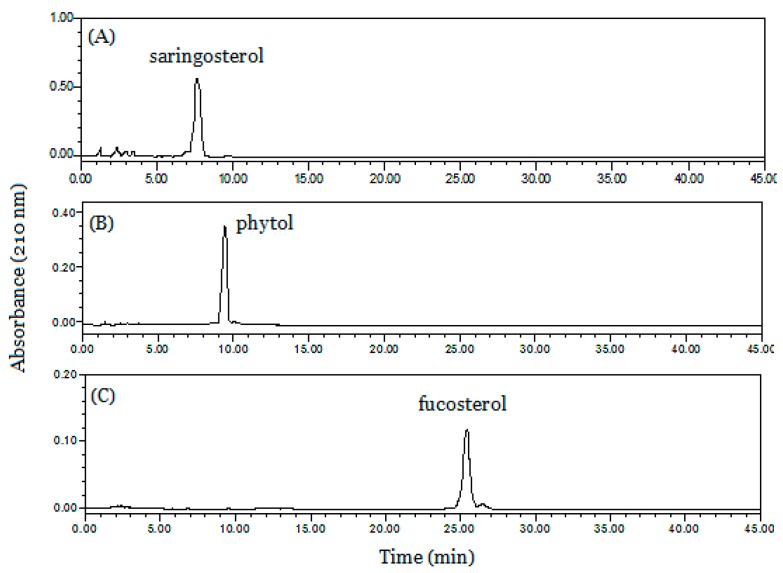
HPLC analysis of obtained HSCCC fractions. (**A**) CCC fraction 1; (**B**) CCC fraction 2; (**C**) CCC fraction 3.

As seen in Figure 4, the entire HSCCC separation process continued for 4 h. Three target fractions could be obtained in 150 min. The eluent corresponding to fractions **1**, **2** and **3** were collected and evaporated, producing 3.1 mg saringosterol, 19.8 mg phytol, and 23.7 mg fucosterol, respectively, in a single-step separation. Then, the target fractions were analyzed by HPLC (shown in Figure 5A–C) and their purities were both above 85%, as determined by HPLC peak areas (the purity of compounds **1**, **2** and **3** were 85.09%, 96.29%, and 93.13%, respectively), which clearly indicate that the two-phase system is very efficient for CCC separation of phytosterols from *S. horneri*.

From the above separation, the advantages of the CCC technique are obvious. First, the use of two-phase solvent systems allows one to choose solvents from an enormous number of possible combinations, making it the most appropriate technique to separate the target compound(s) from a complex sample matrix. Second, it allows sample loads ranging from milligrams to kilograms, depending on the column size. Third, due to the use of biphasic liquid systems, there is no irreversible adsorption caused by a solid support matrix. Thus, the crude *S. horneri* extract can be directly injected for CCC separations with no additional sample pretreatment procedures. This is almost impossible for the preparative HPLC, in which the complex sample matrix always causes contaminations and damage of expensive packing materials, such as reversed-phase silica gel and sephadex gel. In addition, there are practical considerations that make CCC advantageous relative to preparative HPLC. For example, sample solubilization in a biphasic solvent system tends to work very well whereas fairly crude mixtures can be challenging to dissolve in a suitable solvent system for HPLC. Overall, CCC is a promising preparative separation technique, extremely useful for the separation and purification of various natural products.

### 2.5. Structure Elucidation of the Isolated Compounds

The structural identification of compounds **1**–**3** was performed by ^1^H and ^13^C NMR analyses, and the data were compared with literature values. Accordingly, the molecules isolated were identified as saringosterol (**1**) [14,25,26], phytol (**2**) [27,28], and fucosterol (**3**) [14,22,29] (see Supplementary Materials).

## 3. Materials and Methods

### 3.1. Reagents and Materials

Solvents for high-performance liquid chromatography (HPLC) analysis were as follows: acetonitrile and methanol were chromatographic grade and obtained from Merck (Darmstadt, Germany). High-purity water with a resistivity of 18.2 MΩ cm^−1^ was obtained from a Milli-Q water system (Millipore, Bedford, MA). Solvents used for CCC and sample preparation were as follows: methanol, acetonitrile, *n*-hexane, and ethanol were analytical grade and purchased from Huadong Chemicals (Hangzhou, China). The NMR solvent was CDCl_3_ (Fisher Scientific, Loughborough, UK). *S. horneri* were purchased from Dongtou County, Zhejiang Province. The material was identified by Dr. Juanjuan Chen from School of Marine Sciences, Ningbo University, and a voucher specimen was deposited in our laboratory.

### 3.2. Preparation of Crude Extract

*S. horneri* were dried to a constant weight in a vacuum oven at 55 °C and ground into powder for storage in dark conditions. 500 g of the dried material was extracted with 95% ethanol (2 L) at 85 °C for 2.5 h under reflux. The extraction procedure was repeated three times. The extracts were combined and evaporated to dryness by rotary vaporization at 45 °C, yielding 40 g of the crude extract, which was stored in a freezer (−20 °C) for the subsequent separation.

In order to removing chlorophylls and lipids, saponification of crude samples was performed according to the method described by Schröder et al. after slight modification [21]. The 30.0 g of the crude extract was saponated with 100 mL of 10% ethanolic potassium hydroxide solution at 65 °C and stirred for 3 h under nitrogen atmosphere. Then, the solution was cooled, and 100 mL *n*-hexane and 100 mL water were added. The upper phase which contained the unsaponifiable matter was separated. Then the upper organic phase (*n*-hexane phase) was washed with 30% aqueous ethanol until the water phase was almost colorless and the pH was near neutral. After separation, the organic phase was evaporated to dryness by rotary vaporization at 35 °C. The residue (1.365 g) was stored in a refrigerator (−20 °C) for the subsequent CCC separation. Under this storage condition, the extract and its chemical constitutes are stable for at least half a year.

### 3.3. Determination of Solute Partition Coefficient

A suitable partition coefficient (*K*) is usually used as a good sign of the successful CCC separation. The ideal *K* value is usually considered in the range of 0.5 to 2 [18]. The *K* value in the solvent system of target compounds in *S. horneri* was determined by the following method [18]: 2.0 mg crude extract of *S. horneri* was dissolved in 1 mL of the pre-equilibrated solvent system (upper and lower phases 1:1 *v*/*v*) and sonicated to fully dissolved. After two phases separated, 100 μL of each phase was removed and concentrated to dryness. The residue was dissolved in 1 mL methanol and analyzed by HPLC. The *K* value was calculated as the ratio of the peak area of the target compound in the upper phase divided by that in the lower phase.

### 3.4. Preliminary Studies on Retention of the Stationary Phase

The percentage of stationary phase volume retained in the rotating coiled column relative to the total column capacity was determined at various revolutional speeds and flow rate. A series of experiments were conducted with the present CCC apparatus with 140 mL of total capacity in the head-to-tail elution mode. In each series of experiments, the column was first filled with the stationary phase, and then the mobile phase was pumped into the column while it was rotated at a given revolutional speed. After the mobile phase front emerged and hydrodynamic equilibrium state was established in the column, the retention of the stationary phase (*S*_F_) could be determined by displacing the column contents with a flow of nitrogen. Thus, the *S*_F_ was expressed as the ratio of stationary phase retained in the column and the total column capacity.

### 3.5. HSCCC Procedures

CCC separation was accomplished on a type-*J* CCC instrument [30] manufactured by the Zhejiang University machine shop (Hangzhou, China). The CCC apparatus was designed with one 140 mL coil and a counterweight. The multilayer coil was prepared by winding a 45 m × 2.0 mm I.D. PTFE tube for 6 layers. The *β*-value (*β* = *r*/*R*, where r is the distance from the coil to the holder shaft, and *R*, the revolution radius or the distance between the holder axis and central axis of the centrifuge, *R* = 8 cm) varied from 0.33 at the internal terminal to 0.60 at the external terminal.

In this work, the two-phase solvent system is composed of *n*-hexane-acetonitrile-methanol (5:5:6, *v*/*v*). The preparative HSCCC procedures were as follows: the multilayer coiled column was first entirely filled with the upper phase as the stationary phase. The lower phase was then pumped into the head end of the column at a flow rate of 2.0 mL/min, while the apparatus was rotated at 800 rpm. After hydrodynamic equilibrium was reached, 10.0 mL sample solution (samples were dissolved in a 1:1 mixture of upper and lower phase) containing 300 mg crude extract was injected into the separation column through the injection valve. The effluent from the tail end of the separation column was continuously monitored with a UV detector at 210 nm.

### 3.6. HPLC Analysis

HPLC analysis was performed on a Waters Acquity UPLC system (Waters, Milford, MA, USA), which was equipped with a binary pump, a heated column compartment, an autosampler, a PDA detector, and an Empower workstation. The HPLC separation was achieved on a YMC ODS-A HPLC column (150 mm × 4.6 mm i.d., 5 μm, 120 Å) protected with an on-line filter. The mobile phase was composed of methanol/acetonitrile (3:7, *v*/*v*) and isocratic elution mode was employed. The column temperature was set at 25 °C. The flow rate was 0.8 mL/min, and the effluent was monitored at 210 nm.

### 3.7. Nuclear Magnetic Resonance Spectroscopy 

^1^H and ^13^C Nuclear magnetic resonance spectroscopy (NMR) measurements were performed on a Bruker Advance DMX500 spectrometer (Bruker Biospin, Rheinstetten, Germany) at 500 and 125 MHz, respectively. Purified compounds were dissolved in CDCl_3_, and data were processed by WIN-NMR software version 6.1.0.0.

## 4. Conclusions

In conclusion, a high-speed countercurrent chromatographic method was developed and successfully applied to the separation and purification of phytosterols from the crude sample of *S. horneri* using n-hexane-acetonitrile-methanol (5:5:6, *v*/*v*) as the two-phase solvent system. Under the optimum conditions, phytol and two analogue sterols were baseline separated, producing 19.8 mg phytol, 23.7 mg fucosterol and 3.1 mg saringosterol from 300 mg of *S. horneri* extract. Our research demonstrates that HSCCC is a suitable and effective protocol for the preparative separation of sterol analogues from the crude *S. horneri* extract. 

## Figures and Tables

**Table 1:** The *K* values of target compounds in different solvent systems.

Solvent System (*v*/*v*)	*K* Value			Separation Factor (α) ^a^
	Saringosterol (1)	Phytol (2)	Fucosterol (3)	
*n*-hexane-acetonitrile (1:1)	0.97	3.55	4.42	1.25
*n*-hexane-acetonitrile-dichloromethane (10:7:3)	0.79	2.43	2.73	1.12
*n*-hexane-acetonitrile-dichloromethane (8:7:3)	0.68	2.37	2.51	1.06
*n*-hexane-dichloromethane-methanol-acetonitrile (10:3:2:5)	0.43	1.06	1.07	1.01
*n*-hexane-acetonitrile-methanol (5:5:3)	0.94	1.78	2.32	1.30
*n*-hexane-acetonitrile-methanol (5:5:4)	0.84	1.59	2.18	1.37
*n*-hexane-acetonitrile-methanol (5:5:6)	0.72	1.40	2.02	1.44

*^a^* Separation factor of compound **2** and **3** (*α* = *K*_3_/*K*_2_).

## Supplementary Materials

The following are available online at https://www.mdpi.com/1660-3397/17/12/691/s1, Figure S1: ^1^H NMR spectrum of saringosterol (**1**) (CDCl_3_, 500 MHz), Figure S2: ^13^C NMR spectrum of saringosterol (CDCl_3_, 125 MHz), Figure S3: ^1^H NMR spectrum of phytol (**2**) (CDCl_3_, 500 MHz), Figure S4: ^13^C NMR spectrum of phytol (CDCl_3_, 125 MHz), Figure S5: ^1^H NMR spectrum of fucosterol (**3**) (CDCl_3_, 500 MHz), Figure S6: ^13^C NMR spectrum of fucosterol (CDCl_3_, 125 MHz), Table S1: NMR data of isolated compounds in CDCl_3_.
