# Supplementary material for: One-Step Preparative Separation of Phytosterols from Edible Brown Seaweed Sargassum horneri by High-Speed Countercurrent Chromatography
Source: Mar Drugs. 2019 Dec 9;17(12):691. doi: 10.3390/md17120691 (PMC6949986; doi:10.3390/md17120691)
Supplement: Supplementary file 1 [file marinedrugs-17-00691-s001.pdf]

Supplementary Materials

One-Step Preparative Separation of Phytosterols from Edible Brown Seaweeds *Sargassum horneri* by High-Speed Countercurrent Chromatography

Menglu Xia [1], Chunping Liu [1], Lei Gao [2] and Yanbin Lu [1,*]

[1.] Key laboratory of aquatic products processing of Zhejiang Province, Institute of Seafood, Zhejiang Gongshang University, Hangzhou 310035, China; xml429821@163.com (M.X.); lcpzjgsu@163.com (C.L.); luyanbin@zjgsu.edu.cn (Y.L.)

[2.] Hangzhou Nafen BioChem Corporation, Hangzhou 310008, China; gl@zju.edu.cn (L.G.)

* Correspondence: Correspondence: luyanbin@zjgsu.edu.cn (Y.L.); Tel.: +86-571-87103135

Table of Contents page

Figure S1. ^{1}H NMR spectrum of saringosterol (**1**) ($CDCl_3$, 500 MHz) 3

Figure S2. ^{13}C NMR spectrum of saringosterol (**1**) ($CDCl_3$, 125 MHz) 3

Figure S3. ^{1}H NMR spectrum of phytol (**2**) ($CDCl_3$, 500 MHz) 4

Figure S4. ^{13}C NMR spectrum of phytol (**2**) ($CDCl_3$, 125 MHz) 4

Figure S5. ^{1}H NMR spectrum of fucosterol (**3**) ($CDCl_3$, 500 MHz) 5

Figure S6. ^{13}C NMR spectrum of fucosterol (**3**) ($CDCl_3$, 125 MHz) 5

Table S1. NMR data of isolated compounds in $CDCl_3$ 6

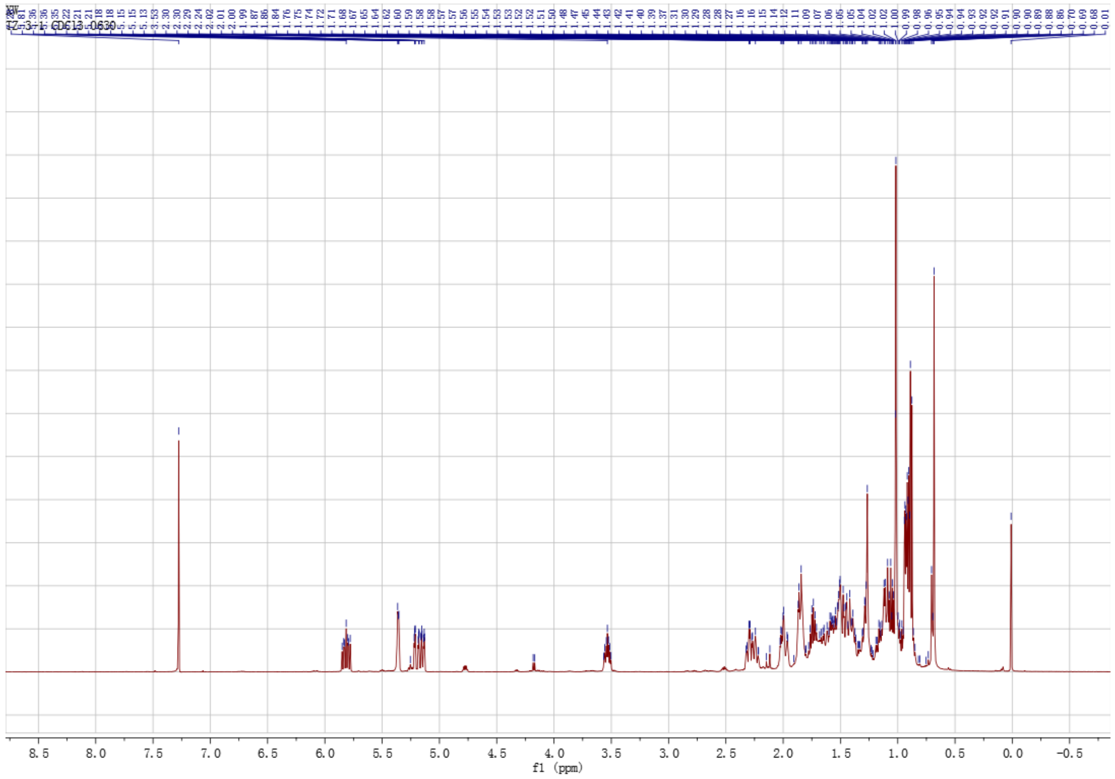

Figure S1. ¹H NMR spectrum of saringosterol (**1**) ($CDCl_3$, 500 MHz).

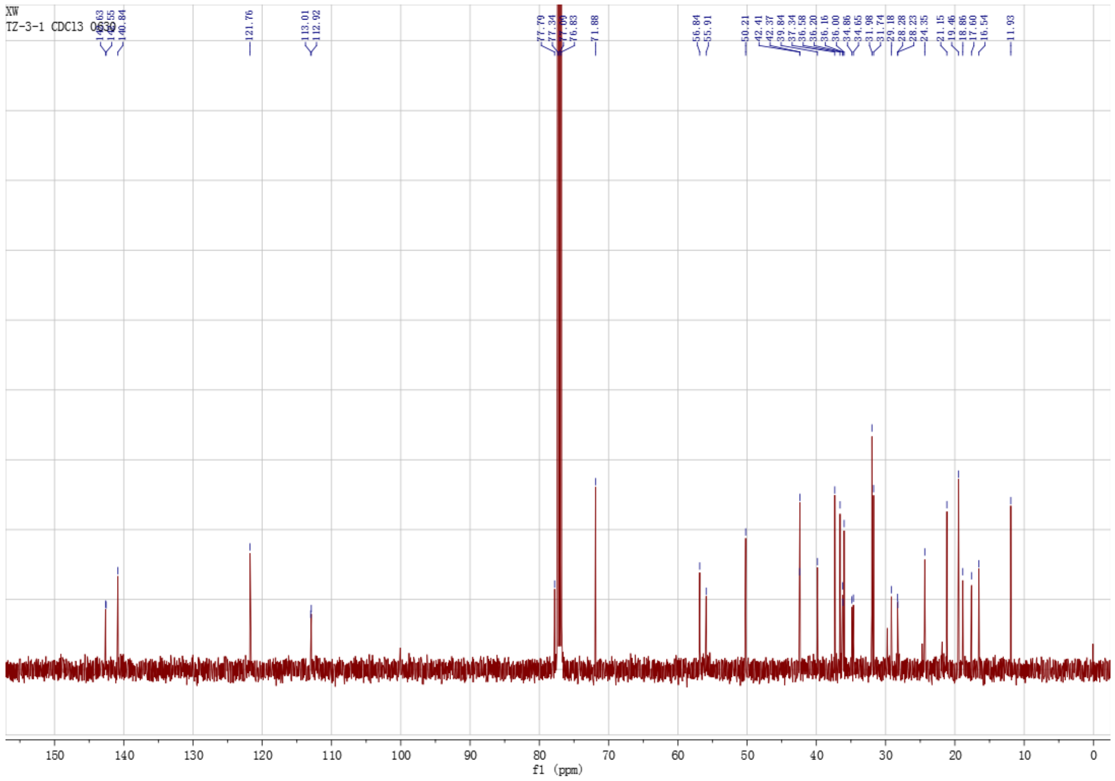

Figure S2. ¹³C NMR spectrum of saringosterol (**1**) ($CDCl_3$, 125 MHz).

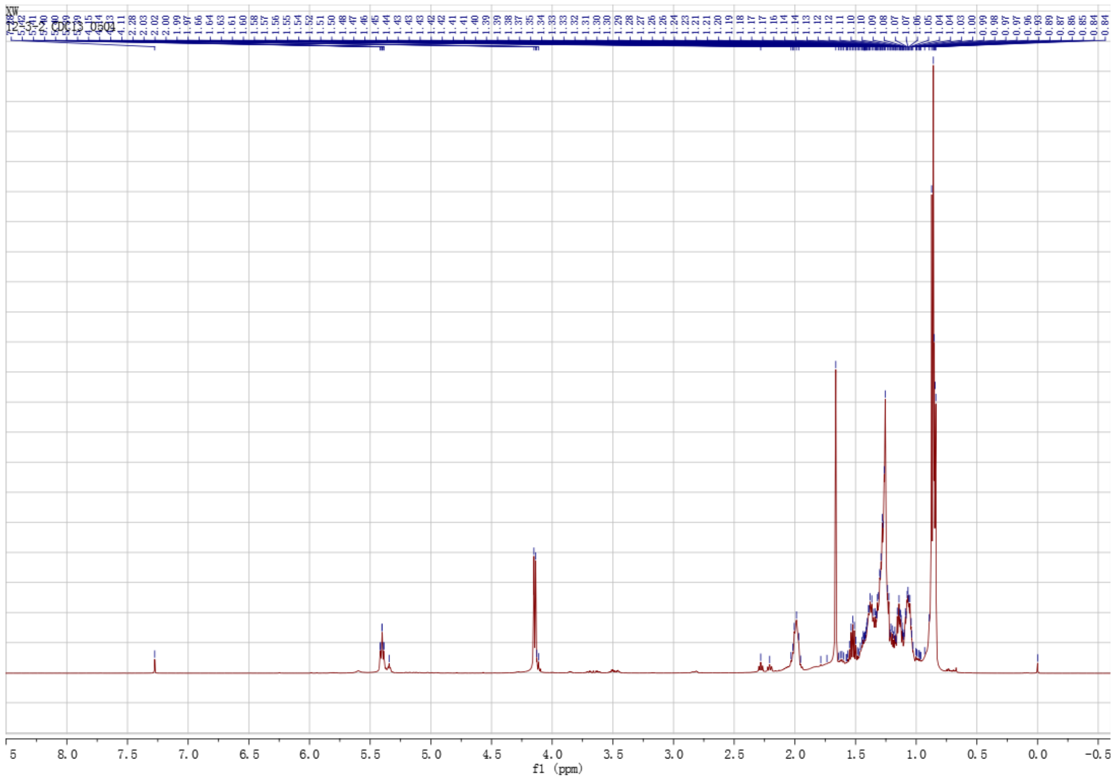

Figure S3. ^{1}H NMR spectrum of phytol (**2**) ($CDCl_3$, 500 MHz).

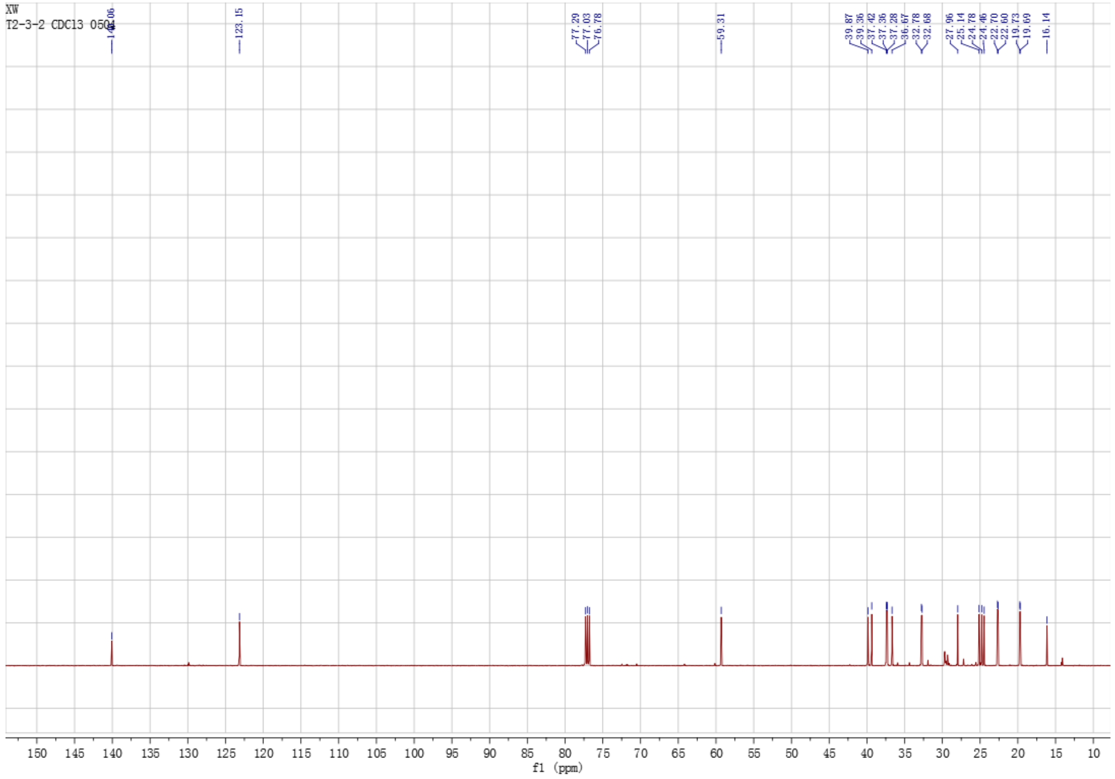

Figure S4. ^{13}C NMR spectrum of phytol (**2**) ($CDCl_3$, 125 MHz).

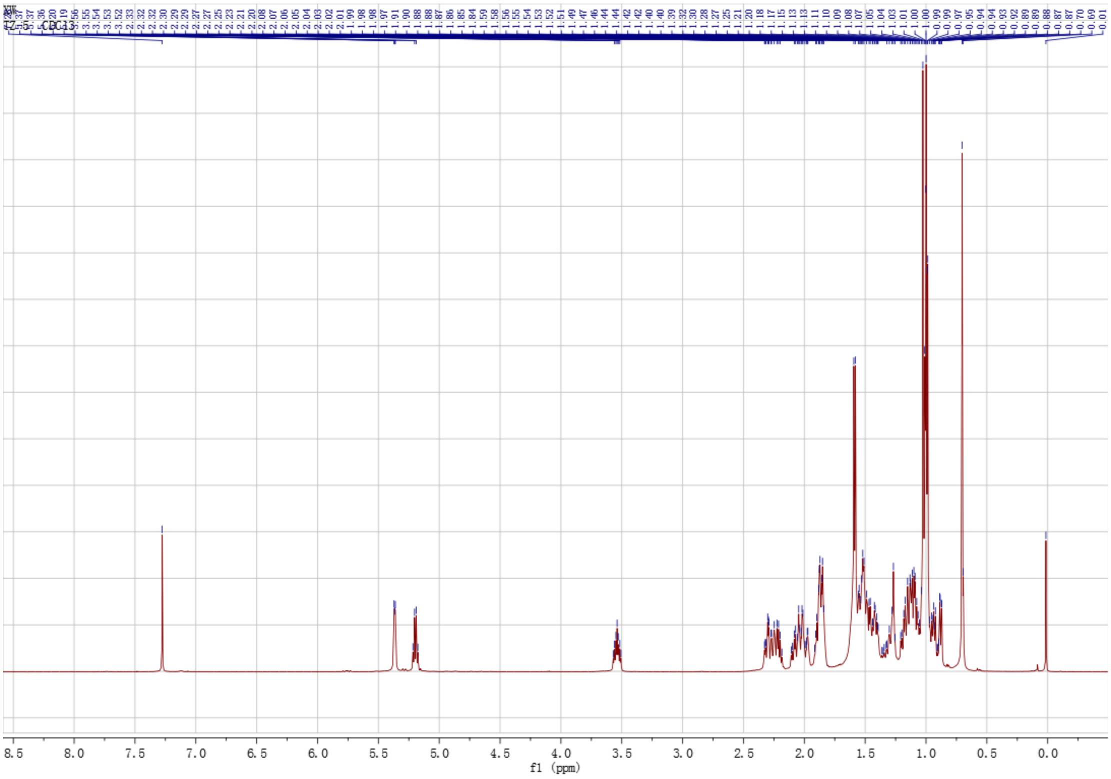

Figure S5. ^{1}H NMR spectrum of fucosterol (**3**) ($CDCl_3$, 500 MHz).

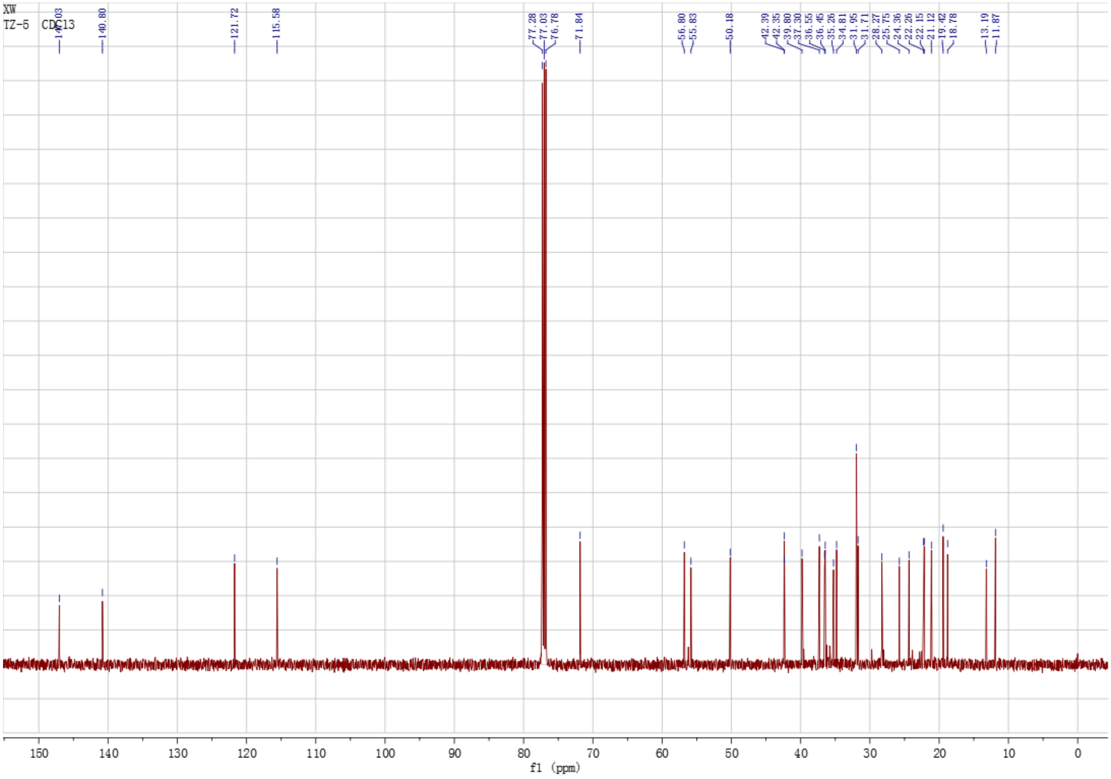

Figure S6. ^{13}C NMR spectrum of fucosterol (**3**) ($CDCl_3$, 125 MHz).

Table S1. NMR data of isolated compounds in $CDCl_3$.

Compounds	^{1}H NMR (500MHz, $CDCl_3$)	^{13}C NMR (125MHz, $CDCl_3$)
Saringosterol (**1**)	5.81 (1H, ddd, J = 17.6, 10.9, 7.0 Hz, H-28), 5.36 (1H, dt, *J*=4.3, 2.0 Hz, H-6), 5.20 (1H, ddd, *J*= 17.4, 3.1, 1.6 Hz, H-29a), 5.15 (1H, ddd, *J* = 10.9, 3.1, 1.5 Hz, H-29b), 3.53 (1H, tt, *J* = 11.0, 4.7 Hz, H-3), 1.02 (3H, s, H-19), 0.95 – 0.93 (3H, m, H-21), 0.91 (3H, dd, *J* = 6.7, 3.5 Hz, H-27), 0.88 (3H, d, *J* = 7.0Hz, H-26), 0.68 (3H, s, H-18)	11.93 (C-18), 16.53 (C-26), 17.60 (C-27), 18.46 (C-21), 19.46 (C-19), 21.15 (C-11), 24.35 (C-15), 28.23 (C-16), 29.18 (C-22), 31.73 (C-2), 31.98 (C-7), 31.98 (C-8), 34.65 (C-23), 35.99 (C-25), 36.16(C-20), 36.58 (C-10), 37.33 (C-1), 42.37 (C-4), 42.40 (C-13), 50.20 (C-9), 55.90 (C-17), 56.83 (C-14), 71.87 (C-3), 77.78 (C-24), 113.00/112.91 (C-29), 121.75 (C-6), 140.83 (C-5), 142.62/142.54 (C-28)
Phytol (**2**)	5.40 (1H, ddt, *J* = 9.7, 6.9, 1.4 Hz, H-2), 4.14 (2H, d, *J* = 6.9 Hz, H-1), 1.66 (3H, d, *J* = 1.3 Hz, H-20), 1.52 (1H, dt, *J* = 13.3, 6.6 Hz, H-15), 0.88-0.83 (12H, m, H-16, 17, 18, 19)	^{13}C NMR (125MHz, $CDCl_3$): 16.14 (C-20), 19.69 (C-19), 19.73 (C-18), 22.60 (C-16), 22.69 (C-17), 24.46 (C-9), 24.78 (C-13), 25.13 (C-5), 27.95 (C-15), 32.68 (C-7), 32.77 (C-11), 36.66 (C-6), 37.28 (C-12), 37.35 (C-10), 37.42(C-8), 39.36 (C-14), 39.86 (C-4), 59.30 (C-1), 123.14 (C-2), 140.04 (C-3)
Fucosterol (**3**)	5.37 (1H, dt, *J* = 4.6, 2.0 Hz, H-6), 5.20 (1H, q, *J* = 6.7 Hz, H-28), 3.54 (1H, tt, *J* = 11.2, 4.7 Hz, H-3), 1.59 (4H, d, *J* = 6.7 Hz), 1.03 (3H, s, H-21), 0.99 (6H, dd, *J* = 6.8, 1.8 Hz, H-19, 26), 0.97 – 0.85 (3H, m, H-27), 0.70 (3H, s, H-18)	11.87 (C-18), 13.19 (C-29), 18.78 (C-21), 19.42 (C-19), 21.12 (C-11), 22.15 (C-26), 24.35 (C-15), 25.75 (C-23), 28.26 (C-16), 31.71 (C-2), 31.94 (C-7), 31.94 (C-8), 34.80 (C-25), 35.25 (C-22), 36.45(C-20), 36.54 (C-10), 37.30 (C-1), 39.80 (C-12), 42.34 (C-4), 42.39 (C-13), 50.17 (C-9), 55.83 (C-17), 56.79 (C-14), 71.83 (C-3), 115.57 (C-28), 121.71 (C-6), 140.79 (C-5), 147.01 (C-24)
